# Joubert Syndrome with Variable Features: Presentation of Two Cases

**Published:** 2013

**Authors:** Mohammad BARZEGAR, Majid MALAKI, Elyar SADEGI-HOKMABADI

**Affiliations:** 1 Professor of Pediatric Neurology, Pediatric Health Research Center, Tabriz University of Medical Sciences, Tabriz, Iran; 2 Assistant Professor of Pediatric Neurology, Pediatric Health Research Center, Tabriz University of Medical Sciences, Tabriz, Iran; 3 Adult Neurologist, Pediatric Health Research Center, Tabriz University of Medical Sciences, Tabriz, Iran

**Keywords:** Joubert Syndrome, Developmental Delay, Respiratory Irregularity, Molar Tooth Sign

## Abstract

Joubert syndrome is a very rare disorder characterized by respiratory irregularities, nystagmus, hypotonia, and global developmental delay with abnormalities of cerebellum. We present two cases of this syndrome with different phenotypes. The first case was an 8-month-old girl with hypotonia, apnea, and mild developmental delay as well as retinal degeneration and unilateral renal cystic dysplasia. The second case was a 27-month-old boy who presented with episodes of hyperpnea, apnea, retinal dystrophy, and severe global developmental delay. Both patients had normal metabolic profile and prototype imaging of joubert syndrome including vermis agenesis and molar tooth sign.

## Introduction

Joubert syndrome (JS) is an autosomal recessive disorder associated with hypoplasia of the cerebellar vermis and the molar tooth sign. It is also associated with hypotonia, ataxia, and characteristic breathing abnormalities (episodic apnea and hyperpnea, global developmental delay, nystagmus, strabismus, and oculomotor apraxia). It may be accompanied by additional features including progressive retinal dysplasia, coloboma, congenital heart disease, microcystic kidney disease, liver fibrosis, polydactyly, tongue protrusion, and soft tissue tumors of the tongue (1-3). There are many suggested presentations for JS besides to what was above-mentioned; but, the molar tooth sign detected by radio-imaging is considered essential for the diagnosis of JS. In this case report, we compare clinical findings of two cases diagnosed with JS in our center during a 4-year period.

## Case Report

**Case 1**

**Fig1(a, b,c,d).**
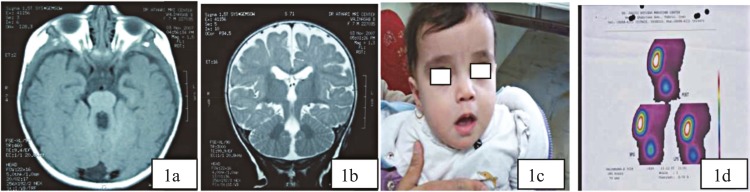
Show imaging finding such as tooth molar sign (1a), vermis agenesis (1b), and face characters including frontal bossing, hypertelorism, bitemporal depression, open mouth, and nystagmus(1c); unilateral renal agenesis is another finding in this case (1d)

The first case was an 8-month-old girl born from non-consanguine parents who presented with apnea following the use of antiepileptic drugs due to suspected seizure. Her cardinal findings were nystagmus, developmental delay (she could not roll in bed, made unknown sounds, could recognize her mother), unilateral dysplastic kidney, and retinal dystrophy. Brain magnetic resonance imaging (MRI) showed typical findings related to Joubert syndrome including vermis agenesis and molar tooth sign which confirmed JS diagnosis in this case (Fig 1a,b,c).

**Case 2**

**Fig2 (a,2b,2c):**
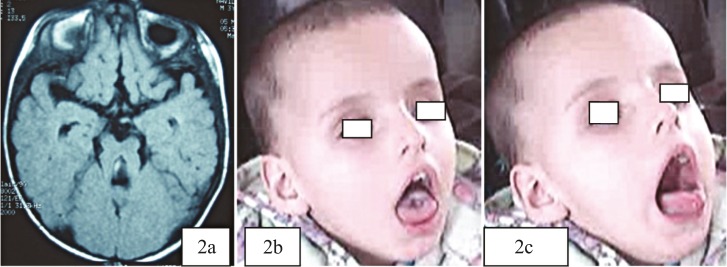
Radio-imaging findings showing molar tooth sign, vermis agenesis, and cortical atrophy (2a); his face characters showing hypertelorism, open mouth, and temporal bone depression (2b); during hyperpnea, he brought out his tongue in panting posture with gazing (2c

A 27-month-old boy was referred to us with severe global developmental delay. He was not able to sit without support and did not interact with his parents. He was born from consanguine parents with no history of such disease in their family. First sibling was completely normal. His cardinal findings were severe hypotonia with normal deep tendon reflexes, episodic hypeventilation and apnea, agitation, tongue protrusion resembling the panting of a dog, and ocular motor apraxia associated with retinal dystrophy in his ophthalmoscopic examination. Kidney function and structure, liver function tests, metabolic screening tests, and echocardiography were all normal. His electroencephalography showed poorly organized sleep features without epileptiform discharges. He showed facial dysmorphism with forehead prominence, deep-set eye, and bilateral epicanthic folds. No limb anomalies such as syndactily or polydactyly were noted. Brain MRI showed typical findings related to JS including vermis agenesis and molar tooth sign (Fig 2 a,b, c). Genetic analysis was not available.

## Discussion

Joubert syndrome is a rare disorder of the cerebellum occurring in 1 of 100,000 live births (3). It may be sporadic or be inherited in the family by an autosomal recessive pattern. The syndrome was first described in 1969 by Marie Joubert and colleagues who reported a 6-monthold infant with an abnormal rapid breathing pattern and developmental delay (4). Since then, approximately 200 additional cases have been published in the literature (5). Ten causative genes have been identified, to date. All these genes encode proteins of the primary cilium including JS in the group of ciliopathiies (6). 

Hyperpnoea in an infant without other signs of respiratory distress may prompt the consideration of JS diagnosis. (5) Typical facial features described for this syndrome include high round eyebrows, broad nasal bridge, anteverted nostrils, and low-set ears. Ocular findings include colobomas, oculomotor apraxia, nystagmus, strabismus, ptosis, and abnormal retinal pigmentation (3,5). They may have an open mouth with a protruding tongue and characteristic rhythmic tongue movements (5). Associated conditions which may involve the eye, the kidney (microcystic renal disease), and the heart (congenital heart disease) are also well described (7). Although the diagnostic criteria for JS have not been established, the clinical features frequently mentioned as essential for diagnosis of classic JS include hypotonia in infancy, developmental delay or mental retardation, and one or both of the following (not absolutely required but helpful): 1- irregular breathing pattern in infancy (intermittent tachypnea and/or apnea), and 2- abnormal eye movements. 

Key neuro-imaging features of JS are hypoplasia of cerebellar vermis and particular midbrain-hindbrain “molar tooth” sign. This sign is considered to be mandatory for the diagnosis of JS. Fetal ultrasound may be useful in at-risk pregnancy allowing the detection of hypoplasia of the cerebellar vermis. With MRI, it is quiet feasible to make the diagnosis before 24 weeks of gestation (8). However, molar tooth sign is not specific for JS. The term Joubert syndrome and related disorders (JSRD) has been recently adopted to describe all disorders presenting the “molar tooth sign” on brain imaging. These disorders are classified into six phenotypic subgroups of pure JS, JS with ocular defect, JS with renal defect, JS with oculorenal defects, JS with hepatic defect, and JS with orofaciodigital defects (9). 

Although our cases had many common findings such as imaging characteristics, they also had common phenotypes such as broad and bulging frontal, open mouth, low-set ears, nystagmus, hypotonia, and developmental delay; however, their presentation was not the same.

First case was from the JS with oculorenal defect subgroup. Unilateral agenesis of the kidney was at first considered to be a co-incidence. This form is characterized by association of neurological signs of JS with both retinal dystrophy and nephronophthisis. About 50% of the patients carry mutations in the CEP290 gene (10). Renal disease may occur in up to 25% of the JS cases mostly attributed to nephronophthisis, a tubulointerstitial disease associated with small cysts at corticomedullary junction that leads to polyuria and polydipsia followed by end stage renal disease in the second decade of life (11). In our first case, unilateral cystic dysplasia was observed in her without any signs supporting NPH (such as polyuria and polydipsia). 

Hyperpnea, apnea, and more severe global developmental delay were more prominent in the second case. Renal structure and function were intact in him. This case was from the JS with ocular defect subgroup. In this subgroup, the neurological features of JS are present in association with retinal dystrophy. The age at onset, progression, and severity are variable. The most common frequently mutated gene in this subgroup is AH11 in almost 20% of cases (13). Respiratory irregularities will be attenuated with ageing up to about six months; however, they may be worsened with emotional stress and using hypnotic drugs (14,15). According to our evaluation, our first case did not show respiratory irregularities except during fever and using hypnotic medications. In the second case, respiratory irregularity was still present in older ages without any special emotional stresses. Several episodes of hyperpnea (lasting almost one minute) ending to apnea for 30 seconds and consequent wake up with fear and irritability occurred each day. 

Both of our cases had retinal dystrophy; but, in the first case, it was due to non-familial sporadic events occurring with renal dysplastic kidney and in the second case, it was associated with normal renal structures. The first patient was born from consanguine parents and it seems that severity of respiratory and cognition problems was more prominent in him. 

**In Conclusion,** Joubert syndrome is not a rare syndrome in our area. It seems to be an under-diagnosed entity. Being familiar with its manifestations and maintaining high index of suspicion are necessary for early diagnosis. 

Neuro-imaging should be done in suspected cases.
